# Characterization of Family D DNA polymerase from *Thermococcus* sp. 9°N

**DOI:** 10.1007/s00792-014-0646-9

**Published:** 2014-05-03

**Authors:** Lucia Greenough, Julie F. Menin, Nirav S. Desai, Zvi Kelman, Andrew F. Gardner

**Affiliations:** 1 New England Biolabs, Inc., 240 County Road, Ipswich, MA 01938 USA; 2 Institute for Bioscience and Biotechnology Research, University of Maryland, National Institute of Standards and Technology, 9600 Gudelsky Drive, Rockville, MD 20850 USA; 3 Present Address: Pfizer Inc., 1 Burtt Road, Andover, MA 01810 USA

**Keywords:** Analytical biochemistry, Archaea, DNA enzymes, DNA polymerase, DNA replication, Family D DNA polymerase, Thermococcus, Replisome, Fidelity

## Abstract

Accurate DNA replication is essential for maintenance of every genome. All archaeal genomes except Crenarchaea, encode for a member of Family B (polB) and Family D (polD) DNA polymerases. Gene deletion studies in *Thermococcus kodakaraensis* and *Methanococcus maripaludis* show that polD is the only essential DNA polymerase in these organisms. Thus, polD may be the primary replicative DNA polymerase for both leading and lagging strand synthesis. To understand this unique archaeal enzyme, we report the biochemical characterization of a heterodimeric polD from *Thermococcus*. PolD contains both DNA polymerase and proofreading 3′–5′ exonuclease activities to ensure efficient and accurate genome duplication. The polD incorporation fidelity was determined for the first time. Despite containing 3′–5′ exonuclease proofreading activity, polD has a relatively high error rate (95 × 10^−5^) compared to polB (19 × 10^−5^) and at least 10-fold higher than the polB DNA polymerases from yeast (polε and polδ) or *Escherichia coli* DNA polIII holoenzyme. The implications of polD fidelity and biochemical properties in leading and lagging strand synthesis are discussed.

**Electronic supplementary material:**

The online version of this article (doi:10.1007/s00792-014-0646-9) contains supplementary material, which is available to authorized users.

## Introduction

Replicative DNA polymerases have evolved to faithfully replicate and maintain genomes to ensure accurate transmission of genetic information. Most DNA polymerases can be classified into families A, B, C and Y according to amino acid sequence similarity to *Escherichia coli* DNA polymerases I, II, III, and IV/V, respectively (Braithwaite and Ito 1993). Eukaryotic DNA polymerases β, λ, μ and terminal transferases comprise family X (Yamtich and Sweasy 2010). Heterodimeric family D DNA polymerases (polD) are unique to archaea and were initially characterized in several *Pyrococcus* species [(Ishino and Ishino 2012) and references therein]. PolD is a heterodimeric enzyme consisting of small (polD-S) and large (polD-L) subunits (Ishino et al. 1998; Cann et al. 1998), together referred to hereafter as polD. PolD possesses both 3′–5′ exonuclease and polymerase activities (Tang et al. 2004; Shen et al. 2003, 2004a, b; Henneke 2012). PolD-S contains a MRE11-like 3′–5′ exonuclease active site and shares limited sequence similarity with several of the small, non-catalytic subunits of the eukaryotic Polδ and Polε (Shen et al. 2004b). A three-dimensional structure of *Pyrococcus horikoshii* (Pho) polD-S N-terminal fragment (1–70 amino acids) shows structural homology to the N-terminal region of B subunits of human DNA Polα and Polε (Yamasaki et al. 2010). Pho polD-L contains amino acids important for polymerase activity and shares sequence similarity with the catalytic subunit of the eukaryotic Polε (Shen et al. 2001; Henneke et al. 2005). The three-dimensional structure of the Pho polD-L N-terminal domain (amino acids 1–300) was solved and reported as essential for protein folding and dimerization (Matsui et al. 2011). In addition, a subset of polD-L contains inteins inserted in conserved amino acid motifs that are spliced during maturation (Perler 2002).

PolD has been proposed as a key replicase in archaea genome replication (Li et al. 2013). Similar to eukarya, a model of archaeal replication proposes that specialized polymerases complete leading [Family B DNA polymerase (polB)] and lagging (polD) strand synthesis (Li et al. 2013). Supporting this model, both polB and polD are required for viability in the archaeon I sp. NRC-1 (Berquist et al. 2007). However, recent gene deletion studies in *Thermococcus kodakarensis* (Tko) and *Methanococcus maripaludis* (Mma) demonstrate that only polD is required for viability and may be the only replicative DNA polymerase required to replicate *both* the leading and lagging strand (Sarmiento et al. 2013; Cubonova et al. 2013). Supporting its essential role in DNA replication, in vivo polD forms complexes with several replication proteins including mini-chromosome maintenance (MCM) helicase, DNA ligase, the archaeal Cdc45 protein and the processivity factor proliferating cell nuclear antigen (PCNA) (Motz et al. 2002; Li et al. 2010, 2011; Kuba et al. 2012).

Despite its proposed essential role in archaeal DNA replication in general and in Tko replication in particular, poor recombinant expression and low solubility have limited study of polD (Jokela et al. 2005). As an alternative to Tko polD, the polD from a closely related organism, *Thermococcus* species 9°N (9°N) was characterized in this study. 9°N was isolated from scrapings of a smoker chimney collected at the 9°N East Pacific Rise vent site, 500 miles south of Acapulco, Mexico at a depth of 2,500 m (Southworth et al. 1996). 9°N polB has been extensively studied (Southworth et al. 1996; Rodriguez et al. 2000) but the essential properties of 9°N polD are not known. Therefore, the aim of this study was to investigate polD biochemical requirements for polymerization, 3′–5′ exonuclease, and incorporation fidelity.

## Materials and methods

### Enzymes

All restriction endonucleases, modifying enzymes, polB [9°Nm DNA polymerase; 9°N/E143D (Southworth et al. 1996)], Gibson Assembly mix, nucleotides, DNA ladders, and expression vectors were from New England Biolabs (NEB, Ipswich, MA, USA).

### PolD cloning and expression

Based on the *Thermococcus* species 9°N genome sequence (data not shown), PCR primers were designed to PCR amplify the polD small and large subunits from genomic DNA (Southworth et al. 1996). To clone by Gibson Assembly (Gibson 2011), sequence overlapping with the cloning vector ends was added to each 5′ end of the forward and reverse PCR primers. PolD-S PCR primers were: polD-S forward: 5′-CTTTAAGAAG GAGATATACA TATGCTGATT GAGGATTTAA TC-3′ and polD-S reverse: 5′-CGGGCTTTGT TAGCAGCCGG TCAAACCCCC TCACAGAACT G-3; (vector sequence for Gibson Assembly is underlined). PolD-L PCR primers were: polD-L forward: 5′-CTTTAAGAAG GAGATATACA TATGGGGGAA GAGCTCTACT CA-3′ and polD-L reverse: 5′-CGGGCTTTGT TAGCAGCCGG CTAACTCCCG AAGAACTCGT C-3′ (vector sequence for Gibson Assembly is underlined). PolD-S and polD-L PCR products were cloned into NdeI and BamHI cleaved vector pAII17 (Perler et al. 1992) by Gibson assembly following the manufacturer’s protocol (New England Biolabs). PolD-S and polD-L gene sequences were verified by DNA sequencing resulting in plasmids pERE1 (polD-S) and pERF5 (polD-L).

### Recombinant polD purification

For polD-S and polD-L expression and purification, NEB T7 Express/pRIL *E. coli* was transformed with either plasmid pERE1 or pERF5. One liter of LB media (10 g tryptone, 5 g yeast extract, 10 g NaCl, 1 g dextrose, 1 g MgCl_2_ per liter, pH adjusted to 7.2) containing 0.1 mg/ml ampicillin was co-inoculated with a single NEB T7 Express/pRIL *E. coli*/pERE1 colony and a single NEB T7 Express/pRIL *E. coli* pERF5 colony and grown at 37 °C to mid-log phase (OD_600_ = 0.5), whereupon protein expression was induced by addition of 0.4 mM β-d-thiogalactopyranoside (IPTG). Cells were then incubated at 37 °C for five hours, and were collected by centrifugation. The cell pellet was suspended in 0.2 L Buffer A (20 mM Tris–HCl, pH 7.5, 0.2 M NaCl, 1 mM EDTA) and lysed by sonication and incubated at 70 °C for 30 min. Cell debris was removed by centrifugation. Supernatant was passed through a 70 ml DEAE column and flow-through was immediately loaded onto a 23-ml Heparin TSK column (pre-equilibrated in Buffer A) and eluted with a Buffer A gradient from 0.2 M to 1 M NaCl. Fractions were collected and assayed for DNA polymerase activity (described below). Peak fractions were loaded onto a HiPrep S100 size exclusion column pre-equilibrated in Buffer A. Fractions were collected and assayed for DNA polymerase activity. Peak fractions were pooled and dialyzed against storage buffer (10 mM Tris–HCl, pH7.5, 100 mM KCl, 0.1 mM EDTA, 50 % glycerol) and stored at −20 °C. The resulting purified polD preparation was clear and lacked color. Protein concentration was determined by absorbance at 280 nm with a NanoDrop spectrophotometer using a molar extinction coefficient of 192,470 cm^−1^ M^−1^. Purified polD was digested into peptides with trypsin and analyzed by LC/MS–MS. Peptide masses matched the protein sequences of polD-S and polD-L with high MS/MS scores (data not shown).

### DNA polymerization activity assay

DNA polymerase activity was measured as previously described (Kong et al. 1993) by incorporation of radioactively labeled nucleotides into an oligonucleotide primed M13mp18 DNA template followed by acid precipitation. In brief, various amounts of polD were incubated with 15 nM primed M13mp18, 0.2 mM each dNTP, 0.2 µCi/µl [α-^32^P]-dCTP, in 1X ThermoPol Buffer (20 mM Tris–HCl, pH 8.8 at 25 °C, 10 mM (NH_4_)_2_SO_4_, 10 mM KCl, 2 mM MgSO_4_, 0.1 % Triton X-100). Reactions were incubated at 65 °C for 30 min, spotted onto 3 mm Whatman filter discs, precipitated and washed with cold 10 % trichloroacetic acid (TCA), and then rinsed with 95 % ethanol and dried under a heat lamp. Incorporated [α-^32^P]-dCTP was quantified using a scintillation counter. Polymerase activity was calculated as the amount of [α-^32^P]-dCTP incorporated (Kong et al. 1993). One unit of polymerase activity was defined as the amount of enzyme that will incorporate 10 nmole of dNTP into acid-insoluble material in 30 min at 65 °C. PolD-S and polD-L alone lack polymerase or 3′–5′ exonuclease activity (data not shown).

To compare polD polymerization from a DNA or RNA primer, substrates were prepared by annealing a DNA oligonucleotide (5′-TTAAGAGGCT GAGACTCCTC AAGAG-3′) or RNA oligonucleotide [5′-(r)UUAAGAGGCU GAGACUCCUC AAGAG-3′] to single-stranded M13mp18 DNA template. The DNA- or RNA-primed M13 was used as a substrate in the DNA polymerase activity assay described above.

To determine the effects of 9°N PCNA and 9°N RFC (hereafter referred to as PCNA and RFC) on polD activity, polD (22 nM) was incubated at 65 °C with PCNA (10 nM) and RFC (21 nM) in a reaction containing 100 µM dATP, dCTP, and TTP, 10 µM dGTP, 2 mM ATP, 0.02 µCi/µl [α-^32^P]-dGTP, 0.5 nM primed M13mp18, 10 mM MgSO_4_, 250 mM NaCl, and 40 mM Tris–HCl, pH 8.0. Aliquots were sampled and reactions stopped by the addition of EDTA (100 mM final concentration). Reactions were analyzed by acid-insoluble counts as described above.

To test the effect of aphidicolin on polB and polD, the DNA polymerase assay described above was repeated in the presence or absence of aphidicolin (Sigma). First, aphidicolin IC50 was determined by measuring DNA polymerization in the presence of increasing concentrations of aphidicolin. In 30 µl reactions, pol B or polD (10 nM) was incubated with primed M13mp18 DNA (15 nM), dNTPs (50 µM), aphidicolin (final concentrations 0, 25, 50, 100, 200, or 400 µM) in 1× ThermoPol buffer for 20 min at 65 °C. Reactions were analyzed by acid precipitation as described above. Aphidicolin inhibition was also monitored during a polymerization time course. In a 150 µl reaction, polB or polD (10 nM) was incubated with primed M13mp18 DNA (15 nM), dNTPs (50 µM) in 1X ThermoPol buffer at 65 °C. After 5 min incubation, aphidicolin (0.2 mM final concentration) or dH_2_O was added to each set of reactions. Aliquots (20 µl) were sampled at 0, 1, 3, 5, 7, 9, 11, 13, and 15 min, and mixed with EDTA (100 mM final concentration). Reaction products were analyzed by acid precipitation as described above.

### 3′–5′ exonuclease assay

The DNA substrate to detect double-stranded DNA-dependent 3′–5′ exonuclease activity was prepared by annealing the 50 nt FAM oligonucleotide (5′-FAM-AGTGAATTCG AGCTCGGTAC CCGGGGATCC TCTAGAGTCG ACCTGCAGGC-3′) to a 67 nt unlabeled template (5′-TTGCTCGTTT GCTGGGAGCC TGCAGGTCGA CTCTAGAGGA TCCCCGGGTA CCGAGCTCGA ATTCACT-3′) as described above. Exonuclease reactions were performed by mixing magnesium-free 1× ThermoPol Buffer II containing 2.0 mM MgSO_4_, FAM-primer-DNA template (15 nM) and polD (10, 5, 2.5, 1.25, or 0.63 nM). Reactions were incubated at 65 °C for 10 min and terminated with EDTA (100 mM final concentration). Reaction products were separated by 15 % TBE-UREA denaturing polyacrylamide gel electrophoresis and quantified using a Typhoon scanner (GE Healthcare).

To calculate rates of 3′–5′ exonuclease activity, FAM-primer-DNA template degradation was monitored over time. Reactions were performed by mixing FAM-primer-DNA template (15 nM) and DNA polymerase (1 nM: polB, polD, polD/D507A, polD/H554A, or polD/D507A/H554A) in 1× ThermoPol buffer and incubated at 65 °C. Aliquots (20 µl) were sampled at various times (0.25, 0.5, 0.75, 1, 2, 3, 4, 5 min) and quenched with EDTA (100 mM final concentration). Products were separated by capillary electrophoresis using a 3730xl DNA Analyzer (Applied Biosystems). Fluorescent peaks were analyzed using Peak Scanner software version 1.0 (Applied Biosystems). Exonuclease product was plotted over time and fit to a linear slope to derive 3′–5′ exonuclease rate (fmol/min).

### PolD 3′–5′ exonuclease-deficient (exo-) mutant construction and purification

The polD 3′–5′ exonuclease domain has been mapped to the small subunit. Previous work identified two polD-S conserved amino acids important for 3′–5′ exonuclease activity: D507 and H554 (Jokela et al. 2004). PolD exonuclease-deficient mutants were constructed by changing either D507, H554, or D507/H554 to alanine by Q5 site-directed mutagenesis (NEB) according to the manufacturer’s recommendations. Oligonucleotides for PolD-S H554A were Forward: TCCGGGCAAC GCCGACGCAG CAC and Reverse: CCAATAAACA TGGTGATATG ATCCGGAACA. Oligonucleotides for PolD-S D507A were Forward: CATTGGCGGTG CTGTCGTGGA CG and Reverse: ATCATATATT TGATGCGGGA AACCAGTTCT TC. Clones were verified by Sanger sequencing and polD-S/D507A, polD-S/H554A or polD-S/D507A/H554A were co-purified with polD-L as described above. “PolD exo-” will refer to the complex of polD-S/H554 and polD-L.

### Fidelity assay

Mutational frequencies and spectra were determined for PCR products amplified by Taq, polD or polD exo-. In this technique, a 619-bp region (nt 2,019–2,637) of single-stranded M13mp18 DNA (NEB) was amplified by PCR, cloned into a linear pUC19 vector by Gibson Assembly, transformed and miniprep DNA was sequenced. PCR reactions (50 µl) were assembled by mixing single-stranded M13mp18 DNA (1 ng/µl), forward primer (0.2 µM), reverse primer (0.2 µM), dNTPs (0.2 mM each) and 1 Unit DNA polymerase in 1× ThermoPol Buffer. Reactions were incubated in a thermocycler with the parameters: 1 cycle at 94 °C for 30 s followed by 25 cycles of denaturation at 94 °C for 15 s, annealing at 55 °C for 15 s and extension at 60 °C for 1 min concluding with one final extension cycle at 65 °C for 10 min. PolD and polD exo- retained 25 % activity after PCR cycling suggesting that sufficient activity remains throughout PCR cycling. Fidelity oligonucleotides used were Forward: CGAGCTCGGT ACCCGGGTTC TCTTGAGGAG TCTCAG and Reverse: ATGACCATGA TTACGCCAGA CGGAAATTAT TCATTAAAG. PCR products were treated with the NEBNext End Repair Module (NEB) to create blunt ends.

A linear pUC19 vector was constructed by inverse PCR. Inverse PCR reactions (50 µl) were assembled by mixing pUC19 (1 ng/µl), forward primer (0.2 µM), reverse primer (0.2 µM), and 25 µl 2× Q5 DNA polymerase Master Mix (NEB). Reactions were incubated in a thermocycler with the parameters: 1 cycle at 98 °C for 10 s followed by 25 cycles of denaturation at 98 °C for 10 s, annealing at 68 °C for 10 s and extension at 72 °C for 15 s concluding with a final extension cycle at 72 °C for 10 min. Inverse PCR primers used were Forward: TGGCGTAATC ATGGTCATAGC and Reverse: CCCGGGTACC GAGCTCGAAT TC.

Fidelity PCR products (0.5 pmol) and linear pUC19 vector (0.5 pmol) in 10 µl dH_2_0 were mixed with 10 µl 2× Gibson Assembly Master Mix (NEB) and incubated at 50 °C for 15 min. NEB 5-alpha Competent *E. coli* were transformed with 1 µl of completed assembly reactions. Miniprep DNA was prepared and sequenced using primers 1224 (CGCCAGGGTT TTCCCAGTCA CGAC) and 1233 (AGCGGATAAC AATTTCACAC AGGA). Sequences were assembled using DNAStar SeqMan Pro software, version 10.1.1 (Madison, WI, USA) and mutations were tabulated. Error rates were calculated as the total number of mutations divided by the total nucleotides sequenced. PolB fidelity was determined by amplification of the β-galactosidase gene (Kermekchiev et al. 2003). Because polB has a lower error rate, sequencing a larger number of nucleotides (>50,000) was required to identify mutations.

## Results

### PolD sequence and phylogeny

Genes encoding polD-S (2,178 bp) and polD-L (5,337 bp) are in a putative operon with the origin recognition protein cdc6. This putative cdc6/polD-S/polD-L operon is downstream of the putative origin of replication conserved among *Pyrococcus* and *Thermococcus* (Myllykallio et al. 2000). 9°N polD-S (726 amino acids; 80.5 kDa) and 9°N polD-L are most similar to Tko polD-S (72 % identity) and polD-L (88 % identity) (Table 1 and Supplementary Figure 1). The polD-L precursor protein (1,765 amino acids; 201.8 kDa) contains a 474 amino acid intein (54.7 kDa) inserted between N962 and D1438 that is spliced during expression to yield mature polD-L (146.9 kDa).

**Table 1:** Comparison of amino acid sequence identity in polD small and large subunits

	9°N polD	
	Small (%)	Large (%)^a^
*Thermococcus kodakaraensis*	72	88
*Pyrococcus furiosus*	51	80
*Methanocaldococcus jannaschii*	37	52
*Methanococcus maripaludis*	41	49
*Haloferax volcanii*	39	44
*Halobacterium* sp. NRC-1	37	51
*Archaeoglobus fulgidus*	41	50
*Thermoplasma acidophilum*	33	41

^a^Mature polD-L subunits lacking an intein

### Requirements for polD polymerase activity

**Fig. 1:**
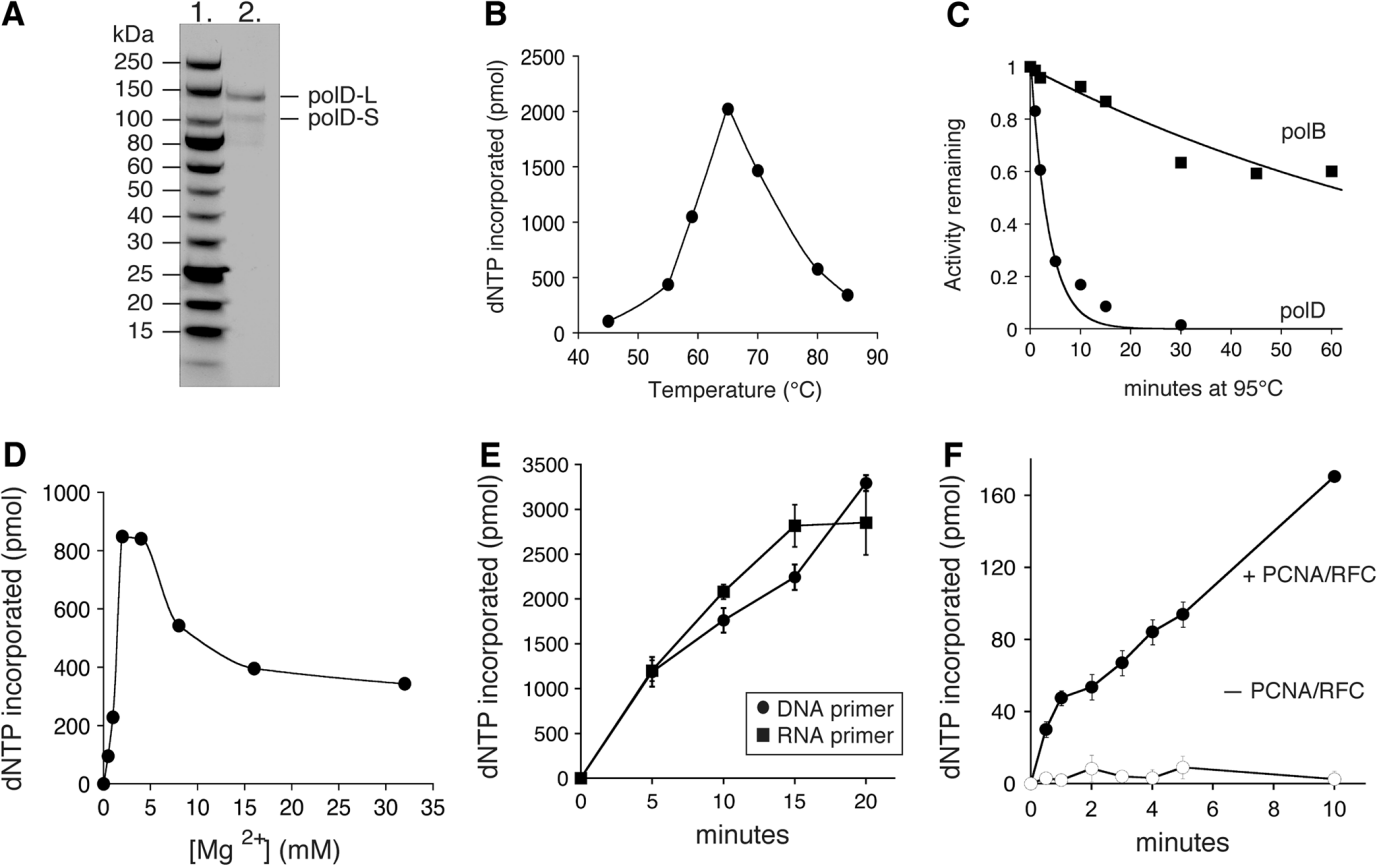
**a** Purified polD was separated by 4–20 % SDS-PAGE and stained with Coomassie blue. *Lane 1* is a Protein Ladder (10–250 kDa) and *Lane 2* is polD. **b**–**f** Nucleotide incorporation by polD into a primed M13mp18 substrate was assayed as described in “Materials and Methods”. **b** PolD temperature optimum. Nucleotide incorporation by polD (10 nM) was measured at various temperatures for 30 min. **c** Heat stability. PolB (10 nM) and polD (10 nM) were incubated at 95 °C in 1× ThermoPol buffer for the indicated times. Nucleotide incorporation by heat-treated polB and polD was then assayed at 65 °C. The fraction of activity remaining was plotted versus incubation time at 95 °C and fit to an exponential equation; polB *filled squares*; polD *filled circles*. **d** PolD Mg^2+^ optimum. Nucleotide incorporation by polD (10 nM) was assayed in 1× ThermoPol II buffer containing various Mg^2+^ concentrations (0.5–32 mM). **e** polD extension from a DNA or RNA primer. Nucleotide incorporation by polD (10 nM) was measured using either DNA- or RNA-primed M13 substrates. DNA-primed M13 substrate *filled circles*; RNA-primed M13 substrate *filled squares*. **f** PCNA stimulates polD. PolD (22 nM) synthesis is stimulated by PCNA and RFC (*filled circles*) compared to a reaction lacking PCNA and RFC (*open circles*)

PolD was purified to homogeneity as described in “Materials and Methods”. Analysis of polD by 4–20 % SDS-PAGE shows two protein bands corresponding to the small and mature large subunits (Fig. 1a). PolD-L intein is spliced during expression to yield a mature polD-L. Because polD-S is highly negatively charged (−55 net charge at pH 7.0), polD-S migrates slower than its calculated molecular weight on an SDS-PAGE gel (Fig. 1a). This observation was also noted for *Pyrococcus abyssi* (Pab) polD-S (Gueguen et al. 2001). PolD optimal polymerase activity is at 65 °C (Fig. 1b). PolD DNA polymerase specific activity on primed M13 substrate is 12,000 units/mg at 65 °C. Its half-life at 95 °C (3 min) is much shorter than polB (67 min) (Fig. 1c) suggesting that interactions with other replisome factors may increase polD thermostability in vivo. Requirements for 9°N polD polymerase activity diverge slightly from other previously described polD from *Pyrococcus* species suggesting diversity among polD active sites. For example, the Mg^2+^ optimum for polD (2–4 mM) (Fig. 1d) is lower than Pab polD (15–20 mM) and Pho polD (17.5 mM) (Gueguen et al. 2001; Shen et al. 2001). Because so few Family D polymerases have been characterized, a general range of optimum activities has not yet been determined.

### PolD extends RNA primers with dNTPs

Replicative DNA polymerases initiate both leading and lagging strand synthesis by extension of a primer with dNTPs. In *Pyrococcus,* a replication model proposes that polD initiates synthesis from RNA primers on the leading strand and is then displaced by polB to complete processive synthesis (Rouillon et al. 2007). Only Pab polD, not polB, can elongate an RNA primer suggesting that Pab polD is required to initiate synthesis from RNA primers during Pab replication (Henneke et al. 2005). In contrast, 9°N polD extends a DNA- or RNA-primed M13 substrate with similar rates (Fig. 1e) while polB initiates from an RNA primer more slowly (data not shown).

### PCNA stimulates polD polymerase activity

Most replicative DNA polymerases on their own have very low processivity. High processivity is achieved by a ring-shaped protein that encircles the DNA and tethers the polymerase to the template for processive DNA synthesis (Indiani and O’Donnell 2006). In the Archaea, the processivity factor is PCNA and it is loaded onto DNA by replication factor C (RFC) (Pan et al. 2011). Both proteins were shown to be essential for cell viability (Sarmiento et al. 2013). Similar to previous studies (Ladner et al. 2011), experiments were carried out in the presence of relatively high salt conditions (0.25 M NaCl) to prevent polD from elongating primed templates in the absence of RFC and PCNA. Therefore, at high salt, polD synthesis was dependent on PCNA binding. Similar to other polDs, DNA synthesis by polD is stimulated 10-fold in the presence of PCNA and RFC likely due to higher processivity (Fig. 1f). Despite stimulation by PCNA and RFC in high salt conditions, the overall polD activity is lower compared to reactions under optimized conditions.

### Aphidicolin inhibits polB but not polD activities

**Fig. 2:**
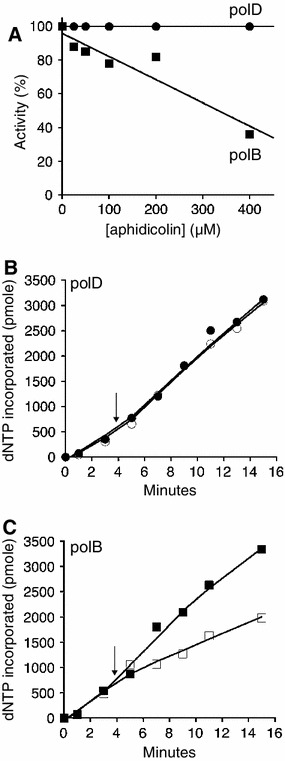
Aphidicolin inhibits polB but not polD synthesis. To test if aphidicolin inhibits polB and polD, DNA synthesis was measured as described in “Materials and Methods”. **a** DNA synthesis by polB (10 nM) (*filled squares*) and polD (10 nM) (*filled circles*) was measured in the presence of increasing concentrations of aphidicolin (0–400 μM) and plotted as a percentage of activity in a reaction without aphidicolin. **b**, **c** DNA synthesis was also measured over a time course. After 4 min of incubation, aphidicolin (final concentration of 200 μM; *open shape*) or dH_2_O (*filled shape*) was added and the reaction was allowed to proceed

Sensitivity to aphidicolin has been used to distinguish DNA polymerase families (Krokan et al. 1981). Aphidicolin inhibits the activity of the eukaryotic family B DNA polymerases by competition with dCTP during DNA synthesis (Dong et al. 1993; Krokan et al. 1981). Increasing concentrations of aphidicolin inhibits polB synthesis (IC50 of 0.3 mM) (Fig. 2a, filled square). In a reaction containing competing dNTPs, polB DNA synthesis slows after the addition of aphidicolin (0.2 mM, Fig. 2c, open square) compared to a parallel reaction lacking aphidicolin (Fig. 2c, filled squares). PolD, on the other hand, is not inhibited by up to 0.4 mM aphidicolin (Fig. 2a, b). *Pyrococcus furiosus* (Pfu) polD and Pab polD are also resistant to aphidicolin inhibition (Gueguen et al. 2001; Ishino et al. 1998). Therefore, although the complete structure of polD is not known, the data suggest that the active site architecture of polD is distinct from polB.

### Determinants of polD 3′–5′ exonuclease activity

During both leading and lagging strand synthesis, the polymerase 3′–5′ exonuclease proofreading activity ensures efficient and accurate genome replication by excising misincorporated nucleotides. Replicative DNA polymerases either encode a 3′–5′ exonuclease domain on the same polypeptide as the polymerase or have associated subunits that provide the exonuclease activity (McHenry 2011). For example, the eukaryotic polδ have both activities located on one polypeptide while in the *E. coli* polIII, two different subunits contain the polymerase and exonuclease activities. Similarly, polB contains both activities in a single subunit, whereas in polD, the large subunit (polD-L) contains the polymerase domain while the small subunit (polD-S) possesses the exonuclease activity.

**Fig. 3:**
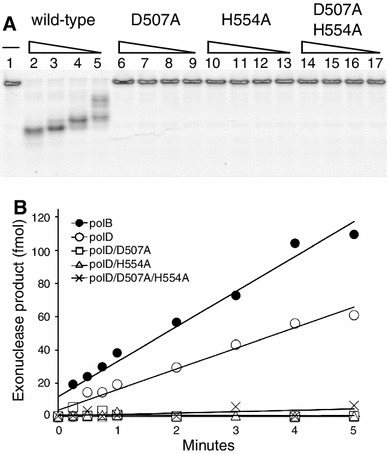
Characterization of polD 3′–5′ exonuclease activity. **a** FAM-labeled primer/template DNA was incubated in 1× ThermoPol buffer. Various concentrations (10, 5, 2.5, 1.25 nM) of polD wild type (*Lanes 2–5*), polD/D507A (*Lanes 6–9*), polD/H554A (*Lanes 10–13*) or polD/D507A/H554A (*Lanes 14–17*) were added. Reactions were incubated at 65 °C for 10 min. Reactions omitting polD were run in parallel (*Lane 1*). Reactions were separated by 15 % TBE-UREA polyacrylamide gel electrophoresis and visualized using a phosphorimager. **b** DNA polymerase (1 nM) was incubated with a FAM-labeled primer-template (15 nM) in 1× ThermoPol buffer. Exonuclease product was quantified over time and fit to a linear slope to derive rates as described in “Materials and Methods”. PolB 3′–5′ exonuclease activity (*filled circle*; 21 fmol/min) was almost two-fold higher than polD (*open circle*; 12 fmol/min). PolD exonuclease-deficient mutants (polD/D507, *open square*; polD/H554A, *open triangle*; and polD/D507A/H554A, *cross hatch*) lacked detectible 3′–5′ exonuclease activity

PolD and polB 3′–5′ exonuclease activities were monitored by degradation of a 5′-FAM-labeled primer/template DNA substrate. PolD 3′–5′ exonuclease activity is dependent on Mg^2+^ (Fig. 3) although Mn^2+^ is a more efficient cofactor (data not shown). Cofactor requirements for 3′–5′ exonuclease activity vary among polDs suggesting a significant diversity of polD active site structures and corresponding functions among the Archaea. For example, 9°N polD and Pho polD 3′–5′ exonuclease activities are dependent on either Mg^2+^ or Mn^2+^ [Fig. 3 and (Shen et al. 2004b)]. In contrast, the *Methanocaldococcus jannaschii* (Mja) polD activity is Mn^2+^ dependent and was not observed in the presence of Mg^2+^ (Jokela et al. 2004). In comparison to polB, the rate of exonuclease activity of polD is about 2-fold lower (21 fmol/min for polB versus 12 fmol/min for polD) (Fig. 3).

Previous studies identified key amino acids in polD-S required for Mja polD 3′–5′ exonuclease activity (Jokela et al. 2004, 2005). Homologous mutations were constructed in 9°N polD-S and 3′–5′ exonuclease activity was tested with Mg^2+^ or Mn^2+^ as the cofactor. PolD-S itself lacks 3′–5′ exonuclease activity (data not shown). PolD D507A, H554A or D507A/H554A variants did not exhibit detectable 3′–5′ exonuclease activity (Fig. 3). Polymerase specific activities of polD exo- mutants were within three-fold of wild type (data not shown).

### PolD fidelity

**Fig. 4:**
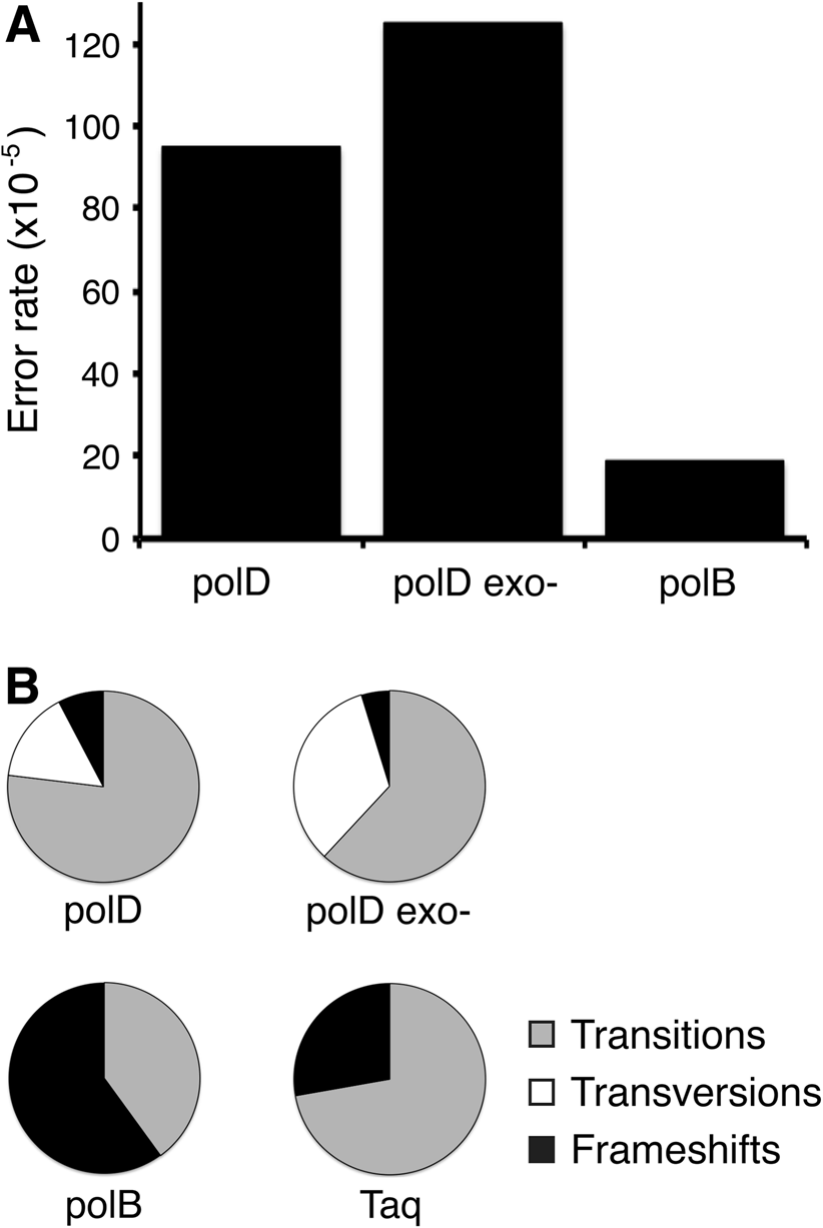
Comparison of error rates and single nucleotide substitutions. **a** PolD (95 × 10^−5^) and polD exo- (125 × 10^−5^) error rates are higher than polB (20 × 10^−5^). **b** Transitions (*gray*) are the majority of single base substitutions during synthesis by polD, polD exo- and Taq DNA polymerases. PolD and polD exo- synthesis also yields transversions (*white*) and frameshift deletions (*black*) at lower frequencies

**Fig. 5:**
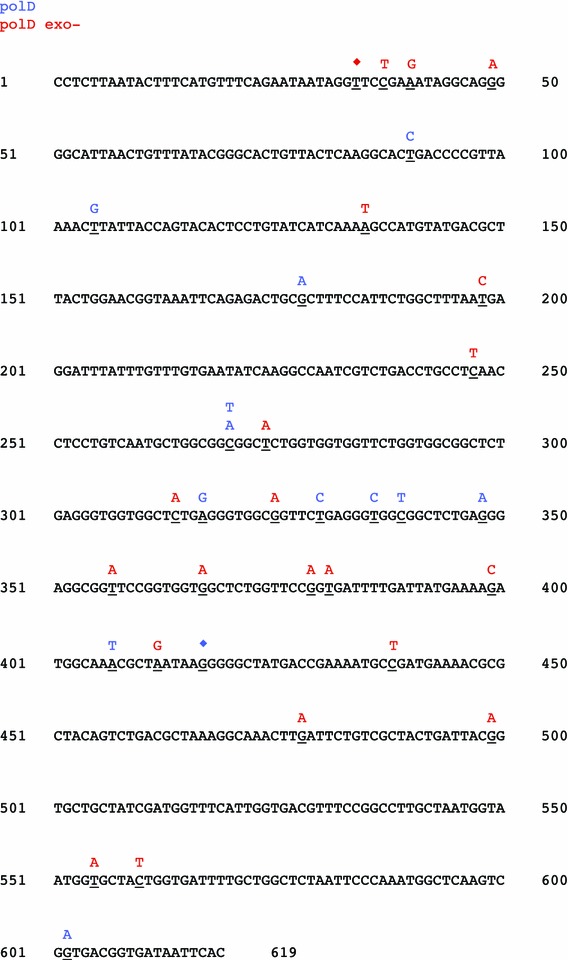
PolD and polD exo- mutational spectra. Types of errors generated by polD (*blue*) and polD exo- (*red*) are shown above the template sequence. Single base deletions are shown by a* filled diamond*

**Fig. 6:**
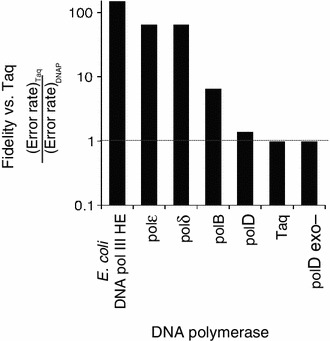
Fidelity of DNA polymerases relative to Taq DNA polymerase. The fidelity of DNA polymerases relative to Taq fidelity was calculated by dividing the [error rate]Taq by the [error rate]DNAP. The error rates of *E. coli* DNA polymerase III holoenzyme (HE) (Pham et al. 1998) and yeast polε and polδ (Shcherbakova et al. 2003) were from previous studies. A *dotted line* represents fidelity equal to Taq DNA polymerase. Values above the line have higher fidelity than Taq while values below the line have lower fidelity

DNA polymerase fidelity is critical for genome integrity. PCR-based fidelity assays were performed to determine the mutational frequency and spectra of Taq, polD, polD exo- (polD/H554A), and polB DNA polymerases. Amplified DNA was sequenced and analyzed to determine the mutational frequency. The error rate was calculated as: error rate = [mutations detected]_total_/[nt sequenced] _total_. The error rate for Taq DNA polymerase (125 × 10^−5^) is in general agreement with published values (Suzuki et al. 1997). Despite 3′–5′ exonuclease proofreading activity, polD error rate (95 × 10^−5^) was only slightly lower than polD exo- (125 × 10^−5^) and Taq DNA polymerase. PolB error rate (19 × 10^−5^) was lower than polD or Taq (Table 2). For polD and Taq DNA polymerases, the majority of mutations observed were transitions while polD and polD exo- also produced transversions (Table 2; Fig. 4). PolD mutations were distributed throughout the amplified DNA fragment rather than clustered in mutational hot spots (Fig. 5). The overall polD and polD exo- fidelities were between one and two orders of magnitude lower than the *E. coli* DNA polIII holoenzyme (HE) (Pham et al. 1998) or Family B DNA polymerases from yeast (polε and polδ) (Shcherbakova et al. 2003) (Fig. 6).

**Table 2:** DNA polymerase nucleotide incorporation fidelity

	Transitions				Transversions								Frameshifts		Total mutations	Total nt sequenced	Error rate^a^ (×10^−5^)
	A > G	T > C	G > A	C > T	G > T	C > A	G > C	C > G	A > C	T > G	A > T	T > A	−1	+1			
polD	1	3	3	2	0	1	0	0	0	1	1	0	1	0	13	13,618	95
polB	0	0	2	2	3	2	0	1	0	0	0	0	0	0	10	50,963	19
polD exo-	2	1	6	4	0	1	1	0	0	0	1	4	1	0	21	16,713	125
Taq	5	5	2	1	0	0	0	0	0	0	0	0	3	2	18	14,237	125

^a^The error rate is calculated as the total number of mutations detected divided by the total number of bp sequenced error rate = [mutations detected]_total_/[nt sequenced]_total_

It has been well documented that reaction temperature and buffer conditions such as pH and divalent metal influence the fidelity of DNA polymerases (Zhang et al. 2009, 2010). A standard set of reaction conditions using ThermoPol buffer was used to be consistent with previous studies on fidelity (Mattila et al. 1991) but may not reflect buffer conditions in vivo. Therefore, polD fidelity in vivo is likely influenced by native buffering conditions.

## Discussion

PolD is an essential DNA polymerase required for viability in *Thermococcus* *kodakaraensis* and *Methanococcus maripaludis* (Sarmiento et al. 2013; Cubonova et al. 2013). Despite its proposed role in replication, study of polD has been limited by poor recombinant expression and low solubility (Jokela et al. 2005). A well-expressed and soluble polD from *Thermococcus* species 9°N facilitated a detailed biochemical analysis of family D DNA polymerase and its role in replication. PolD contains both DNA polymerase and proofreading 3′–5′ exonuclease activities to ensure efficient and accurate genome replication. This study has characterized the polymerase and exonuclease activities of polD and report, for the first time, the fidelity of a member of Family D DNA polymerases.

During chromosomal replication, only correctly base paired nucleotides are in the proper geometry for efficient incorporation by the DNA polymerase. However, if an incorrect nucleotide is incorporated into the newly synthesized strand, then the structural perturbation caused by the mispair creates a kinetic barrier to further extension and polymerization stalls. The primer strand then shifts from the polymerase domain to a separate 3′–5′ exonuclease active site where misincorporated dNMP is hydrolyzed in the 3′–5′ direction. On average, this proofreading 3′–5′ exonuclease activity improves DNA polymerase fidelity by 2- to 100-fold (Kunkel 2009).

The DNA polymerase exonuclease is generally proposed to proceed via a two-metal mechanism (Derbyshire et al. 1991; Beese and Steitz 1991). In Family A and B DNA polymerases, two essential divalent metal ions (A and B) are bound in the exonuclease active site and play key roles in substrate binding and catalysis (Joyce and Steitz 1995). Metal A interacts with the phosphate oxygen atoms of the 3′ dNMP and is bound to the protein by acidic amino acids in the exonuclease active site. Metal A polarizes a water molecule for nucleophilic attack on the 3′-dNMP phosphodiester bond. Metal B is hypothesized to stabilize the transition state or intermediate. In Family A and B DNA polymerases, mutating conserved Exo I, II, or III motif acidic amino acids reduces 3′–5′ exonuclease activity by disrupting coordination of Metals A and B (Reha-Krantz 2010). Similarly, mutating conserved amino acids (D507A or H554A) abolishes polD exonuclease activity. Therefore, D507 or H554 may coordinate Metal A or B in the polD exonuclease active site. Further biochemical and structural studies will help elucidate the molecular determinants of the polD 3′–5′ exonuclease active site and how exonuclease and polymerase domains are coordinated to detect and remove misincorporated nucleotides during synthesis.

One requirement of a replicative DNA polymerase is high fidelity of DNA synthesis to ensure accurate duplication of the genome. PolD has been proposed to be the only replicative DNA polymerase in Tko and Mma (Sarmiento et al. 2013; Cubonova et al. 2013) and the lagging polymerase in other archaeal species (Henneke et al. 2005). Surprisingly, despite the presence of a 3′–5′ exonuclease proofreading activity, polD has a relatively high error rate compared to polB and other replicative DNA polymerases.

Based on elevated polD error rates observed in vitro, one would expect that many mutations would be introduced during replication in vivo. However, spontaneous mutation rates in polB-deletion strains (Tko ΔpolB) that only encode polD are similar to wild type suggesting that other factors may increase polD incorporation fidelity in vivo (Cubonova et al. 2013). PolB and polD incorporation fidelities may offer insight into roles in leading and lagging strand synthesis in the archaea. Differences in leading and lagging strand mutation rates have been observed in *E. coli* where the lagging strand is copied with higher fidelity than the leading strand (Fijalkowska et al. 1998). On the leading strand, processive DNA polymerases favor mismatch extension without dissociation. Lagging strand polymerases dissociate from mismatches during Okazaki maturation and are repaired. Based on this model, one would predict leading strand synthesis is completed by polD with lower fidelity while polB is responsible for higher fidelity lagging strand synthesis. Such a model may occur in archaea but will require further testing with a reconstituted archaeal replisome to measure fidelity during coordinated leading and lagging strand synthesis.

It is likely that additional replisome factors such as PCNA or GINS may increase polD incorporation fidelity in vivo by increasing base selection fidelity, stimulating 3′–5′ exonuclease proofreading, or lowering mismatch extension rates. By analogy, PCNA binding to polB acts as a switch between the polymerase and exonuclease modes to modulate 3′–5′ exonuclease activity (Mayanagi et al. 2011). As a result, PCNA-dependent exonuclease activity may increase polB fidelity leading to low error rates in vivo. Similar interactions may modulate polD fidelity. Subsequent kinetic studies will examine polD base selection and extension from mismatches as well as the effect of accessory factors on polD fidelity to have a more comprehensive understanding of replication in the Archaea.

## Electronic supplementary material

Below is the link to the electronic supplementary material.

Supplementary material 1 (PDF 239 kb)
