# Supplementary material for: Characterization of Family D DNA polymerase from Thermococcus sp. 9°N
Source: Extremophiles. 2014 May 3;18(4):653–64. doi: 10.1007/s00792-014-0646-9 (PMC4065339; doi:10.1007/s00792-014-0646-9)
Supplement: Supplementary file 1 — Supplementary material 1 (PDF 239 kb) [file 792_2014_646_MOESM1_ESM.pdf]

A. polD-S multiple sequence alignment

```
T. species 9°N        --MLIEDLIKNKYLITPSAYYLLEPHYKR-DFTLAELIKFAKARGTFVID 47
T. kodakaraensis      --MLVEDLLKNNYLITPSAYYLLSDHYKK-AFTLAELIKFAKNRGTFVVD 47
P. furiosus           MDEFVKSLLKANYLITPSAYYLLREYYEKGEFSIVELVKFARSRESYIIT 50
M. jannaschii         -MEIINKFLDLEALLSPTVYEKLKNFDEEKLKRLIQKIREFKKYNNAFIL 49
M. maripaludis        ---MINDFLNIQVLLAPESYSRLQNLDSNDFSDLFNKIKEFKSKKDDFIL 47
H. volcanii           --MPLETPARIVRTLVGRGYNAEREAVTLIAGSDDPGRTLAR-------- 40
H. species NRC-1      --------------------------------------------------
A. fulgidus           -----MVIKNIDAATVAKKFLVRGYNIDPKAAELICKSGLFS-------- 37
T. acidophilum        -----------MVIWFFYGFRKIMHLS---LKRIIVKCKMS--------- 27

T. species 9°N        SSIAEAFLTEKGLFSTG----ELTEQTPLEVSEE------EPLEASREEI 87
T. kodakaraensis      SNLAREFLAEKGIISSGNGALDGSVLSGISVSESPVKPNGEAVLESESDE 97
P. furiosus           DALATEFLKVKG-------------------------------------- 62
M. jannaschii         LDEKFLDIFLQKDLDEI--------------------------------- 66
M. maripaludis        LDSHFLNIFLGNNLKNI--------------------------------- 64
H. volcanii           --------------------------------------------------
H. species NRC-1      --------------------------------------------------
A. fulgidus           --------------------------------------------------
T. acidophilum        --------------------------------------------------

T. species 9°N        HGDFAESAEIPVSETVTASRPEEIPSSEDFESAEASSSISTGDVVESEVA 137
T. kodakaraensis      SGAIESSLQEGVEDGVESIAEPSAASVEDVVVAGESSLASEGSSLEDEIN 147
P. furiosus           --------------------------------------------LEPILP 68
M. jannaschii         --------------------------------------------------
M. maripaludis        --------------------------------------------------
H. volcanii           --------------------------------------------------
H. species NRC-1      --------------------------------------------------
A. fulgidus           --------------------------------------------------
T. acidophilum        --------------------------------------------------

T. species 9°N        TASSGEESVEP-AEIIG-----ETSISTGTAPELEQSLTLNSVEEPVSGE 181
T. kodakaraensis      TEPELSSSMDDGAEVVQSSEGDETSISTGNALGSVDYGGSDSESEALEEA 197
P. furiosus           VETKGG------------------FVSTGESQKEQSYEESFGTKEEISQE 100
M. jannaschii         -------------------------INEYKDFDFIFYYTGEEEKEKPKEV 91
M. maripaludis        -------------------------FEEYGKFDFLAYYTSNEVASEEKII 89
H. volcanii           -------------------------VVEAAPDDALRITADHVREVLASA 64
H. species NRC-1      --------------------------------------------------
A. fulgidus           ----------------------------DELVDKICRIANGGFIIEKSVV 59
T. acidophilum        ---------------------------QLDRTSLIQYFNSNGVLISPEAL 50

T. species 9°N        ISEGESFVSTGTPEHEELVDEAAGSLDSSLTEVVEDSLPVEAEPLNGNGY 231
T. kodakaraensis      SSGGESFISTGTPEDEALLEESPEGEFPDENGFSDESLPVENG--SENGY 245
P. furiosus           IKEGESFISTGS---EPLEEELN-------------SIGIEEIG------ 128
M. jannaschii         KK-EIKKETEEKIEKEKIEFVKKEEKE----------------------- 117
M. maripaludis        EKDDFEPVNEVKLENEEIIEPKRENK------------------------ 115
H. volcanii           PAADPSGTEPTP-------------------------------------- 76
H. species NRC-1      --------------------------------------------------
A. fulgidus           EEFLRNLSN----------------------------------------- 68
T. acidophilum        DK-LIKYSSSSLIED----------------------------------- 64

T. species 9°N        GNYTDSEEYYENGENGNGVKPKIVYGDYGIPIAYVG-EDVEGEKSYSTYE 280
T. kodakaraensis      G---DSEEYYENGDN--GVKPKIVYGDYGVPIAYVADETPEEEKAYSTYS 290
P. furiosus           ----ANEELVSNGND--NGGEAIVFDKYGYPMVYAPEEIEVEEKEYSKYE 172
M. jannaschii         ---------------------QFIKKSDEDVEEKLKQLISKEEKKEDFDA 146
M. maripaludis        ----------------------VYDLDFEDIEEEEPELDEIHKKLE---E 140
H. volcanii           --------------------PAAADAANAADADPAASAASDPSVSAATSH 106
H. species NRC-1      --------------------------------------------------
A. fulgidus           ----------------------LKPATLTPRPEE---------------- 80
T. acidophilum        ----------------------LIKNSDGYIDEK---------------- 76

T. species 9°N        DVVIKPKEGFHYRAKEIPDEWELTFDVKNVKFEAPKVKNAASKEGEIIIQ 330
T. kodakaraensis      DLVIAPKEGFHYRAKEIPDEWELAFDVKNVKFEVPKVKNAQSKEGEVIIQ 340
P. furiosus           DLTIPMNPDFNY--VEIKEDYDVVFDVRNVKLKPPKVKNGNGKEGEIIVE 220
M. jannaschii         ERAKRYEHITKKIKESVNSRIKWIAKDIDAVIEIYEDSDVSGKSTCTGTIE 196
M. maripaludis        ERKERILEVRSIRESVNNKINYYAKDIDAQIHVYDEYDVTGKSTCEGTLD 190
H. volcanii           DNAAHTDQSAPAEAQGTPGGRNVDPALRSLEIAN---DMTGQSTGTGEYD 153
H. species NRC-1      --------------------------------------MTGASTGTGEYQ 12
A. fulgidus           ----RKVEEVKAS----------------CIALKVIKDITGKSSCQGNVE 110
T. acidophilum        -YVERYVSRPKVRN---------------DYEVYLP-DVR----FNASIE 105
```

:

```
T. species 9°N       AYSSYFKSRLRKMRRIFRENPEIGTVIDIAKLSYVRDDE-VTIIGLVNDK 379
T. kodakaraensis     AYSSYFKSRLKKMRRIFRENPEIGTIVDIAKLSYVREDD-VTIIGLVNEK 389
P. furiosus          AYASLFRSRLKKLRKILRENPELDNVVDIGKLKYVKEDETVTIIGLVNSK 270
M. jannaschii        DFVKYFRDRFERLKVFIERKAQRKGYPLKDIKKMKGQKD-IFVVGIVSDV 245
M. maripaludis       DFVKYFKDRCSTLRKIIELKNQKKAYPLNQLFRRKKENE-VFIAGIVSDV 239
H. volcanii          DFVKVFRDRYEKLSKLLRGRVNHRPAKAISNMSGGEDAE---LIGLVDDV 200
H. species NRC-1     DFVSVFRDRYDRLAAQLRGRVNHRPTSALASMPGGSDAA---IVGMVNDI 59
A. fulgidus          DFLMYFNSRLEKLSRIIRSRVNTTP----IAHAGKVRGN-VSVVGMVNEV 155
T. acidophilum       DFRKMFVSKYEKLSKIITSSSSMRG-SISIKTAKRSQGE-VKVVGMVSEV 153
```

```
T. species 9°N       RETAKG-YMFEVEDATGRVKVFLGRDKENASQAYNAIMPDSVVAFRG--- 425
T. kodakaraensis     RETRKG-YLFEIEDATGRIKVFIGSDKEGANEAYSTIMPDSVVAFRG--- 435
P. furiosus          REVNKG-LIFEIEDLTGKVKVFLPKDSEDYREAF-KVLPDAVVAFKG--- 315
M. jannaschii        DSTRNGNLIVRIEDTEDEATLILPKEKIEAGKIPDDILLDEVIGAIGTVS 295
M. maripaludis       NSTKNGHKMVELEDENGTFRVLLMKDKMEKGDLPSDVLLDEVIGFEGTIN 289
H. volcanii          RSTKSGHWIIDLEDTTGTFPCLVMKDKDIAGYVDELLMDECIAVQGT--- 247
H. species NRC-1     RSTTSGHWRVELEDTNGVFPVLVLKDRDVSDLVDDLLLDEVIAVSGT--- 106
A. fulgidus          YE-RGDKCYIRLEDTTGTITCVATGKN---AEVARELLGDEVIGVTG-LL 200
T. acidophilum       SMTKNGHKRIVIEDLDDSITAIVMKD---RGPVNEIILEDEVIGIIGSVS 200
```

```
T. species 9°N       QPG-RGIFFANRVFLPDVPKFRREKPPLEEKVYAILLSDIHVGSNKFCEK 474
T. kodakaraensis     TPG-KGIFFANRVFLPDVPKFKRSKPPLEEKVYAILLSDIHVGSNKFCEE 484
P. furiosus          VYSKRGILYANKFYLPDVPLYRRQKPPLEEKVYAILISDIHVGSKEFCEN 365
M. jannaschii        KSGS--SIYVDEIIRPALP-PKEP-KRIDEEIYMAFLSDIHVGSKEFLHK 341
M. maripaludis       DKGD--LMFVDRAFRPDIT-PKPT-KTTDEKIYTAFLSDVHVGSREFLNK 335
H. volcanii          LSGDAGILFVDSMHFPDVPRSHRSN-TADRDVQAALISDVHVGSQEFMAD 296
H. species NRC-1     LADDGTILFADDIYFPEVPRTYSPS-TADRSVQAALISDVHVGSQEFAAD 155
A. fulgidus          KGSS---LYANRIVFPDVP-INGN-GEKKRDFYIVFLSDTHFGSKEFLEK 245
T. acidophilum       QSSKDPVIFVNEIIRPDIP-YRVIDEEKHEPVYVASISDIHVGSKTFRKN 249
```

```
T. species 9°N       AFEKFLEWLNGEVNSRAEEELVSRIKYMIIGGDVVDGVGIYPGQYNELAI 524
T. kodakaraensis     AFIKFLEWLNGEVNSRTEEELVSRIKYIIIGGDVVDGVGIYPGQYNELAI 534
P. furiosus          AFIKFLEWLNGNVETKEEEEIVSRVKYLIIAGDVVDGVGVYPGQYADLTI 415
M. jannaschii        EFEKFIRFLNGDVDNELEEKVVSRLKYICIAGDLVDGVGVYPGQEEDLYE 391
M. maripaludis       VFAKFIRFLNGEVNNGLEEKIVSRLKYISIAGDLVDGVGIYPGQEYDLYD 385
H. volcanii          AWNRFADWLHTEEAAR--------IEYLLIAGDMVEGVGVYPNQDEELDV 338
H. species NRC-1     AWRSFADWLHTPAAES--------VEYLLIAGDMVEGVGVYPGQDEELDI 197
A. fulgidus          EWEMFVRWLKGEVGGKKSQNLAEKVKYIVIAGDIVDGIGVYPGQEDDLAI 295
T. acidophilum       EFEAMVRWISGSDPD------ASRVKYLILSGDVVDGIGVYPDQENDLEI 293
```

```
T. species 9°N       PDIFDQYEALANLLRNVPDHITMFIGPGNHDAARTALPQPGFYEEYAKPI 574
T. kodakaraensis     PDIFDQYEALANLLKQVPDHITMFIGPGNHDAARTALPQPGFYEEYAKPL 584
P. furiosus          PDIFDQYEALANLLSHVPKHITMFIAPGNHDAARQAIPQPEFYKEYAKPI 465
M. jannaschii        VDIIEQYREIAMYLDQIPEHISIIISPGNHDAVRPAEPQPKLPEKITK-L 440
M. maripaludis       VDIISQYAEVAAYLEQVPEHIKVIICPGNHDALRPAEPQPTFDESITS-L 434
H. volcanii          IDIYEQYEAFSEHLKSVPGDLEIVMIPGNHDAVRLAEPQPGFDDELRD-I 387
H. species NRC-1     VDIYDQYETFAEHLKDVPGDMEIVMIPGNHDAVRLAEPQPAFDEELRS-I 246
A. fulgidus          SDIYGQYEFAASHLDEIPKEIKIIVSPGNHDAVRQAEPQPAFEGEIRS-L 344
T. acidophilum       LNPLEQYANLAEYLVDVPEDVKVFVMPGNHDTVRLSEPQPVFPGKIRD-L 342
```

```
T. species 9°N       YKLKNAVIISNPAVINLHGREFLIAHGRGIEDVVDFIPG--RTHHKPAEA 622
T. kodakaraensis     YKLKNAVIISNPAVIRLHGRDFLVAHGRGIEDVVDFVPN--RSHHRPAEA 632
P. furiosus          YKLKNAVIISNPAVIRLHGRDFLIAHGRGIEDVVGSVPG--LTHHKPGLP 513
M. jannaschii        FNRDNIYFVGNPCTLNIHGFDTLLYHGRSFDDLVGQIRA--ASYENPVTI 488
M. maripaludis       FPKENVTFVGNPGVVNMHGLDLLMYHGRSFDDIIGQISS--AQYTNPPSI 482
H. volcanii          MSAHDARITSNPSMVTLEGVNVLMYHGVSLDEVIAELPADKASYDEPHKA 437
H. species NRC-1     MRAHDARITSNPSTVTIDGVSVLLYHGVSLDEVIAEHPSDDVTYDDPQNA 296
A. fulgidus          FPK-NVEHVGNPAYVDIEGVKVLIYHGRSIDDIISKIPR--LSYDEPQKV 391
T. acidophilum       FEP-NVAFLPNPYNLKLEGKNVLVYHGMSLNDMIELIPG--ANYDSIGKA 389
```

```
T. species 9°N       MVDLLKLRHLAPTFGNKVPIAPDPEDTLVIESVPDLFQAGHVHVMEYRIY 672
T. kodakaraensis     MVELLKLRHIAPTFGNKVPIAPDPEDTLVIESVPDLFQAGHVHVMQYKTY 682
P. furiosus          MVELLKMRHVAPMFGGKVPIAPDPEDLLVIEEVPDVVHMGHVHVYDAVVY 563
M. jannaschii        MKELIKRRLLCPTYGGRCPIAPEHKDYLVIDRDIDILHTGHIHINGYGIY 538
M. maripaludis       MTELLKRRHLCPTYGGRCPIAPEHVDYLAIHTEPDIFHTGHIHINGYDNY 532
H. volcanii          MYQLLKKRHVAPQFGGHTRLAPEELDYLVMEDVPDIFHTGHVHKLGWGKY 487
H. species NRC-1     MELLLKKRHVAPPFGGRTRLAPEAEDHLAIDTVPDVFHTGHVHKLGVGIH 346
A. fulgidus          MEELLKRRHLSPIYGGRTPLAPEREDYLVIEDVPDILHCGHIHTYGTGFY 441
T. acidophilum       IEAILVRRHLSPKYGGNTPMIPSAVDYHVIEEVPDIFITGHIHSHYIGNY 439
```

```
T. species 9°N       NGVFVINSGTWQAQTEFQKMVNIIPTPARVPIIDVETARLRAVVSFDQFC 722
T. kodakaraensis     NGVFVINTGTWQAQTEFQKMVNIIPTPARVPIIDVETARLRAVVRFDQFC 732
P. furiosus          RGVQLVNSATWQAQTEFQKMVNIVPTPAKVPVVDIDTAKVVKVLDFSGWC 613
```

```
M. jannaschii          RGVVMVNSGTFQEQTDFQKRMGISPTPAIVPIINMAKVGEKGHYLEWDRG 588
M. maripaludis         HGVRMINSGTFQEQTDFQKRMGIKPTPGIIPIQDLSKREQ--HMIEWNQG 580
H. volcanii            HNVLAVNSGCWQAQTDFQKSVNIDPDAGYAPIVDLDTLNMTVRKFS---- 533
H. species NRC-1       HNVRLVNSGCWQHQTAFQESVNISPDVATAPILDLDTLDITVHKFS---- 392
A. fulgidus            RGVFMVNSSTWQAQTEFQKKVNLNPMPGNVAVYRPGGEVIR---LRFYGE 488
T. acidophilum         KGVRYVNSSTWQSQTEYQKMMNFNPKPSKLTLFDLYSRSV--IVKDFDTT 487

T. species 9°N         EGV--- 725
T. kodakaraensis       EGV--- 735
P. furiosus            ------
M. jannaschii          VLEVRY 594
M. maripaludis         KIEIN- 585
H. volcanii            ------
H. species NRC-1       ------
A. fulgidus            ------
T. acidophilum         L----- 488
```

B. polD-L multiple sequence alignment

```
T. species 9°N         ---MGEELYSPEMKAYFESLQRE---------------------IDRAYE 26
T. kodakaraensis       ---MSEEIYSPEMKAYFESLQRE---------------------IDRAYA 26
P. furiosus            ------MELPKEIEEYFEMLQRE---------------------IDKAYE 23
M. jannaschii          MIVMVHVACSENMKKYFENIVDE---------------------VKKIYR 29
M. maripaludis         ---MLHVSASKGMTEYFKNILDD---------------------VSNLYN 26
H. volcanii            --------MREEETRYFRRIEAR---------------------LDEAFD 21
H. species NRC-1       --------MRPTDETYFETLETG---------------------LDDAFE 21
A. fulgidus            --------MDATLDRFFPLFESESNEDFWRIEEIRRYHESLMVELDRIYR 42
T. acidophilum         -----MSQKPFDLEGYRKYITEK---------------------VREAFN 24

T. species 9°N         IARKARSQGKDPSLDVEVPQATDMAGRVESLVGPPGVAERIRELV-KEYG 75
T. kodakaraensis       IARKARAQGKDPSFDVEVPQATDMAGRVESLVGPPGVAERIRELV-KEYG 75
P. furiosus            IAKKARSQGKDPSTDVEIPQATDMAGRVESLVGPPGVAQRIRELL-KEYD 72
M. jannaschii          IAEECRKKGFDPTDEVEIPLAADMADRVEGLVGPKGVAERIRELV-KELG 78
M. maripaludis         LAEECRKNGYDVTDHVEIPLAKDMADRVEGIVGPKNVAERIRELV-SEFG 75
H. volcanii            LAEAAKATGYDPKTEVEIPVAKDMADRVENILGIDGVAERVRELE-GEMS 70
H. species NRC-1       VAEAARERGEDPTPNVEIPVAKDMADRVENILGIDGVAERVRDLD-GEMS 70
A. fulgidus            IAEAARKKGLDPELSVEIPIAKNMAERVEKLMNLQGLAKRIMELEEGGLS 92
T. acidophilum         VAQEARAKGLDVSDHVEIPLASDMAERIEALIGIKGIAQEIRDLS-SRMS 73

T. species 9°N         KELAALKVVDEIIEGKFGDLGSKEKYAEQAVRTALAILTEGIVSAPLEGI 125
T. kodakaraensis       KEIAALKVVDEIIEGKFGDLGSKEKYAEQAVRTALAILTEGIVSAPLEGI 125
P. furiosus            KEIVALKIVDEIIEGKFGDFGSKEKYAEQAVRTALAILTEGIVSAPLEGI 122
M. jannaschii          KEPAALEIAKEIVEGKFGNFD-KEKKAEQAVRTALAVLTEGIVAAPLEGI 127
M. maripaludis         KEPAALEIAKEIVEGKFGEFG-REVGAEQAVRTALAVITEGIVAAPLEGI 124
H. volcanii            REEAALELVTDFVDGNVGDYDSREGKVEGAVRTAVALLTEGVVAAPIEGI 120
H. species NRC-1       REEAALELVADFVDGRVGDYDTDAGKIEGAVRTAVALLTEGVVAAPIEGI 120
A. fulgidus            RELICFKVADEIVEGKFGEMPKEE-AIDKAVRTAVAIMTEGVVAAPIEGI 141
T. acidophilum         REEVSLEMSRRIAA---MFKDNRKEALDKAIRVGLAILTEGILVAPLEGI 120

T. species 9°N         ADVKIKRNTWADNSEYLALYYAGPIRSSGGTAQALSVLVGDYVRKKLGLD 175
T. kodakaraensis       ADVKIKRNEWADGSEYLALYYAGPIRSSGGTAQALSVLVGDYVRRKLGLD 175
P. furiosus            ADVKIKRNTWADNSEYLALYYAGPIRSSGGTAQALSVLVGDYVRRKLGLD 172
M. jannaschii          ADVKIKKN--PDGTEYLAIYYAGPIRSAGGTAQALSVLVGDFVRKAMGLD 175
M. maripaludis         AYVKIKKN--SDNSEYLAIYFAGPIRSAGGTAQALAVLVGDYVRKNMGLD 172
H. volcanii            DRVEILEN--DDGTEFVNVYYAGPIRSAGGTAQALSVLVADYARSLLDID 168
H. species NRC-1       DRVEVNDN--DDGTQYVAVYYAGPIRSAGGTAQALSVLVADYARAMLGID 168
A. fulgidus            ARVRIDRE--N----FLRVYYAGPIRSAGGTAQVISVLVADYVRRKAEIG 185
T. acidophilum         ADVYIGKN--QDGSEYVGISYAGPIRGAGGTAQALSVLIGDVVRRELGIS 168

T. species 9°N         RFKPSEEHIERMVEEIDLYHRAVTRLQYHPEADEVRLAMRNIPIEITGEE 225
T. kodakaraensis       RFKPSDEHIERMVEEVDLYHRAVTRLQYHPEADEVRLAMRNIPIEITGEE 225
P. furiosus            RFKPSGKHIERMVEEVDLYHRAVSRLQYHPSPDEVRLAMRNIPIEITGEA 222
M. jannaschii          RYKPTEDEIERYVEEVELYQSEVGSFQYNPTADEIRTAIRNIPIEITGEA 225
M. maripaludis         RFKPTEDEVERYGEEVDLYQSEVTTFQYQPKAEEIRVAVRNISVEITGEA 222
H. volcanii            EYKARTDEVERYVEEINLYDKETG-LQYSPKDKESRFIAENMPIMLDGEA 217
H. species NRC-1       AFKPRDDEIERYAEEVDLYDSETG-LQYSPKDAETTFITEHCPVMLDGEA 217
A. fulgidus            RYVPTEEEILRYCEEIPLYKKVAN-LQYLPSDEEIRLIVSNCPICIDGEP 234
T. acidophilum         RFQPTEDEIERYIEEIESYDRIKH-LQYMPTPDEIKLVVRNSPICIDGEG 217
```

```
T. species 9°N      TDKVEVS-HRNVPGVETNHLRGGAILVLAEGVLQKAKKLVKYIDKMGVEG 274
T. kodakaraensis    TDKVEVS-HRNVPGVETNHLRGGAILVLAEGVLQKAKKLVKYIDKMGIEG 274
P. furiosus         TDDVEVS-HRDVEGVETNQLRGGAILVLAEGVLQKAKKLVKYIDKMGIDG 271
M. jannaschii       TDDVEVSGHRDLPRVETNQLRGGALLVLVEGVLLKAPKILRHVDKLGIEG 275
M. maripaludis      TDDVEVSGHRDLPRVETNQIRGGALLALVEGVLLKAPKILRHVDKLNIEG 272
H. volcanii         TGDEEVSGYRDLERVDTNSARGGMCLVMAEGIALKAPKIQRYTRQLDEVD 267
H. species NRC-1    TGNEEVDGFRDLERIDTNSPRGGMCLVLAEGIALKAPKIQRYTRNLDEVA 267
A. fulgidus         TESAEVSGYRNLPRVETNRVRGGMALVIAEGIALKAPKLKKMVDEVGIEG 284
T. acidophilum      SEEEEVSGHRDMERIKTNRIRGGMCLVLCEGLVQKARKILKYTSSMHLDD 267

T. species 9°N      WDWIKEFVEAKEKGKSSEEPKADESKAEDSGAR--EEVAEKVEKGFYYEL 322
T. kodakaraensis    WDWIKEFVEAKEKGKSSEE-NKDESKAEDTGTESVAEKKENVEKGFYYEL 323
P. furiosus         WEWLKEFVEAKEKGEEIEE---SESKAEESKVET----RVEVEKGFYYKL 314
M. jannaschii       WDWLKDLMSKKEEKEEKDEKVDDEEID-----------EEEEEISGYWRD 315
M. maripaludis      WNWLKELKSKKEEVIEELEE--ENDEYN----------YEDEEDLSQYED 310
H. volcanii         WPWLQDLIDGTIGKDDDNAANADDAGDDGD--------EAEAETDPDAEA 309
H. species NRC-1    WPWLQDLIDGTIG-ADDADEDTPDAGSDSD--------ATDEGDAPSAST 308
A. fulgidus         WEWLDALIK----------------GGGDSG--------SEEEK------- 304
T. acidophilum      WNFLANLGGKAEGKSS---------------------------------- 283

T. species 9°N      YERF------RANIAPNKKYTKEIIGGRPLFAEPSENGGFRLRYGRSRVS 366
T. kodakaraensis    YEKF------RANIAPNKKYTKEIIGGRPLFAEPSTNGGFRLRYGRSRVS 367
P. furiosus         YEKF------RAEIAPSEKYAKEIIGGRPLFAGPSENGGFRLRYGRSRVS 358
M. jannaschii       VK-----------IEANKKFISEVIAGRPVFAHPSKVGGFRLRYGRSRNT 354
M. maripaludis      YE-----------VEAVTKFIGEVIAGRPVFSHPSKKGGFRLRYGRSRNT 349
H. volcanii         DDAESDAPDGPTRVEPATKFLRDLIAGRPVFGHPSAPGGFRLRYGRARNH 359
H. species NRC-1    DAEE------PPRAAPSDKFLRDLIAGRPVFGHPSKNGGFRLRYGRARNH 352
A. fulgidus         -----------AVIKPKDKYLSDIVAGRPVLSHPSRKGGFRLRYGRARNS 343
T. acidophilum      --------------KKSDKFLKDIVAGRPVFSHPSRPGGFRLRYGRSRVS 319

T. species 9°N      GFATWSVNPATMLILDEFIAIGTQMKTERPGKGCIVTPATTVEGPIVKLK 416
T. kodakaraensis    GFATWSVNPATMLILDEFIAIGTQMKTERPGKGCIVTPATTVEGPIVRLK 417
P. furiosus         GFATWSINPATMVLVDEFLAIGTQMKTERPGKGAVVTPATTAEGPIVKLK 408
M. jannaschii       GFATQGFHPALMYLVDEFMAVGTQLKTERPGKATCVVPVDSIEPPIVKLK 404
M. maripaludis      GFATDGFHPAIMYLVDDFMAVGTQLKTERPGKATCVVPVDSIEGPIVKLN 399
H. volcanii         GFATAGVHPATMHIVDDFIATGTQIKTERPGKAGGVVPVDSIEGPTVRLA 409
H. species NRC-1    GNATAGVHPATMHLLDDFLATGTQIKTERPGKAAGIVPVDSIEGPTVKLA 402
A. fulgidus         GFATVGVNPATMYLL-EFVAVGTQLKVERPGKAGGVVPVSTIEGPTVRLK 392
T. acidophilum      GLAAASLNPATMYIMGKFIAIGSQIKVELPGKAAAVTPCDTIDGPTVLLK 369

T. species 9°N      DGSVVRVDDYETALRVKNEIEEILYVGDALVNFGDFVENNQTLLPANYVE 466
T. kodakaraensis    NGSVVRVDDYETALKVRNEVDEILYVGDALVNFGDFVENNQTLLPANYVE 467
P. furiosus         DGSVVRVDDYNLALKIRDEVEEILYLGDAIIAFGDFVENNQTLLPANYVE 458
M. jannaschii       NGDVIRVDTIEKAMDVRNRVEEILFLGDVLVNYGDFLENNHPLLPSCWCE 454
M. maripaludis      DGSVLKIDTVEKAKQYTDEVQEILFLGDILVNYGDFLENNHTVLPSSWCT 449
H. volcanii         NGDVRRIDDPEEAKELQNGVEKILDLGEYLVNFGEPVENNHPLAPASYVF 459
H. species NRC-1    NGDVRHINDPADALDVRNGVAEILDVGEYLVNYGEFVENNHELAPASYAP 452
A. fulgidus         NGDVVKINTLSEAKALKGEVAAILDLGEILINYGDFLENNHPLIPASYTY 442
T. acidophilum      NGDHVKINDIEKAREVYDDVVETTDAGEILIAYGDFLENNYPLPTPSFTV 419

T. species 9°N      EWWVQELVKAIEELYEVELKPFSDNPREAVEEAAEYLEVDPDFLESLLRD 516
T. kodakaraensis    EWWVQELVQAIKDLYEVELQPFAENDREAVEEAAEYLEVDPDFLWNLLKD 517
P. furiosus         EWWIQEFVKAVNEAYEVELRPFEENPRESVEEAAEYLEVDPEFLAKMLYD 508
M. jannaschii       EWYEKILI-------------------------ANNIEYDKDFIK----- 474
M. maripaludis      EWYEKILK-------------------------SQNLEYTEEFIK----- 469
H. volcanii         EWWIQEFEATEAN--------------------VQALRDDPAVDLE---- 485
H. species NRC-1    EWWIQDLDAAGAD--------------------VQALRDSPYVDLT---- 478
A. fulgidus         EWWIQEAEKAG-------------------------LRG----DYR---- 459
T. acidophilum      EWWEQYLPDGVNAK------------------------------------ 433

T. species 9°N      PLRVKPSVELAIHLSKVLDIPFHPYYTLYWNTLKPEEVEELQKALLN-AQ 565
T. kodakaraensis    PLRVKPDVETAIHLSTVLDIPFHPYYTLYWNTLQPEEVEELQKALLG-AQ 566
P. furiosus         PLRVKPPVELAIHFSEILEIPLHPYYTLYWNTVNPKDVERLWGVLKDKAT 558
M. jannaschii       ----NPKPEEAVKFALETKTPLHPRFTYHWHDVSKEDIILLRNWLLK--- 517
M. maripaludis      ----NPGQNEAVNYAKITKTPLHPKYTYFWHDISKENIFSLRSWVIG--- 512
H. volcanii         ----EPSVEQALSWATEFDAPLHPVYTYLWHDISVERFDALADAVAAGEI 531
H. species NRC-1    ----APTADQAMAWATDYDAPLHPAYTYLWHDIDVEQFRALADAVADAHT 524
A. fulgidus         ----KISEEEALKLCDEFHVPLHPDYTYLWHDISVEDYRYLRNFVSDNGK 505
T. acidophilum      ----DIDQFSAVEISRKYGIPLHPYYDYYWHDISFEDLEFLVKNAEQWSI 479
```

```
T. species 9°N        IEWDEFRKL----------KFAKRVILENDPQ--IKRYLELLGLPHRLER 603
T. kodakaraensis      IEWAEFRKN----------RFAKKVVLENDKN--IKRYLELLGLPHRLER 604
P. furiosus           IEWGTFRGI----------KFAKKIEISLDDLGSLKRTLELLGLPHTVRE 598
M. jannaschii         -GKEDSLEG----------KKVWIVDLEIEEDKKAKRILELIGCCHLVRN 556
M. maripaludis        -GKYNPSND----------SWELNYDPEDEEILNAKRYLELIGCPHTVME 551
H. volcanii           VAAEADGGTTAALEHDNEPEHGLEGTLVLDNAPEIREALEHLLVAHRQTD 581
H. species NRC-1      DASDDDD---------------RGVLVLDHTTTVRETLEAALLVTHHQGD 558
A. fulgidus           IEGKHGK-----------------SVLLLPYDSRVKEILEALLLEHKVRE 538
T. acidophilum        TEDGMRVPY------------------------PAFDVFIRLGIEFRRSG 505

T. species 9°N        TEDRRKVIVIDYPWSSALLTPLGN---------LEWEFKAKPFFTVIDII 644
T. kodakaraensis      VEKKRKVIVVEYPWSAALLTPLGN---------LEWEFKAKPFYTVIDII 645
P. furiosus           G-----IVVVDYPWSAALLTPLGN---------LEWEFKAKPFYTVIDII 634
M. jannaschii         K---KVIIEEYYPLLYSLGFDVENK----KDLVENIEKILESAKNSMHLI 599
M. maripaludis        E---KVEIFEYYPFLYSLGYDFDEK----RDMIDNIDEKLQNTKNNMHFI 594
H. volcanii           E---ALRVPVWRPLARSLGLTDDRERTWELDDLSERARTWDDGDNAVEAV 628
H. species NRC-1      D---TIRVDDWLPLARSLGVTESLDREWET--LSEAAAEWP---NAVRAV 600
A. fulgidus           S---FIVIETWRAFIRCLGLDEKLSKVSEVS-----------GKDVLEIV 574
T. acidophilum        D---YLIIRDYYPLLISLGYDVRNGK-------IVNVKKYERKGSVMETV 545

T. species 9°N        NESNPIKLRDRGISWIGARMGRPEKAKERKMKPPVQVLFPIGLAGGQSRD 694
T. kodakaraensis      NENNRIKLRDRGISWIGARMGRPEKAKERKMKPPVQVLFPIGLAGGQSRD 695
P. furiosus           NENNQIKLRDRGISWIGARMGRPEKAKERKMKPPVQVLFPIGLAGGSSRD 684
M. jannaschii         NLLAPFEVRRNTYVYVGARMGRPEKAAPRKMKPPVNGLFPIGNAGGQVRL 649
M. maripaludis        NTIAPFEIRRNAYIYVGARMGRPEKAAARKMKPPVNGLFPIGNAGALVRL 644
H. volcanii           NEVAPFNVRERAPTRIGNRMGRPEKSERRDLSPAVHTLFPIGEAGGSQRD 678
H. species NRC-1      NEVAPFSVQERAPTRIGNRMGRPEKSESRDLSPAVHTLFPIGDAGGSQRD 650
A. fulgidus           NGISGIKVRPKALSRIGARMGRPEKAKERKMSPPPHILFPVGMAGGNTRD 624
T. acidophilum        NYLSGLIIKPRAPTRVGSRLGRPEKAGDRKMKPMVHSLFPVESYGEARRS 595

T. species 9°N        IKKAAEEG-------KTAKVEIAFFKCPKCGHTGPEHLCPVCGTRKELLW 737
T. kodakaraensis      IKKAAEEG-------KTARVEIAFFKCPKCGHVGPEHLCPVCGTRKELLW 738
P. furiosus           IKKAAEEG-------KIAEVEIAFFKCPKCGHVGPETLCPECGIRKELIW 727
M. jannaschii         INKAVEEN-------NTDDVDVSYTRCPNCGKISLYRVCPFCGT------ 686
M. maripaludis        INKAVEEG-------KTDEIEIANVKCS-CGKVSLYRTCPFCGN------ 680
H. volcanii           VGDAARHR-GESGKRGQISVRLGQRKCPDCGAFGFKSKCPDCGGHTEPHY 727
H. species NRC-1      VADAARYAPDMSDTPGEIPVRVGDRVCPSCDEHTYESRCPDCGDWTDPHY 700
A. fulgidus           IKNAINYTKSYNAKKGEIEVEIAIRKCPQCGKETFWLKCDVCGELTEQLY 674
T. acidophilum        IIGANKNS------EGSYKAEVFFYRCNSCGFETPTPVCPRCGG------ 633

T. species 9°N        HCPKCNVDYPESEAKEFGFRCPRCDVELKPYAEREIKPSELLRRAMDNVK 787
T. kodakaraensis      HCPKCGADYPESDAKDFNYRCPKCDVELKPYAEREIKPADLLRQAMDNVK 788
P. furiosus           TCPKCGAEYTNSQAEGYSYSCPKCNVKLKPFTKRKIKPSELLNRAMENVK 777
M. jannaschii         ------------------------KVELDNFGRIKAPLKDYWYAALKRLG 712
M. maripaludis        ------------------------SVEPTGPSRIKLPIKDYWYKTLENLK 706
H. volcanii           ECDDCG--SVIEPDESGRVYCERCEWDVESAEWQDVDLNSEYRDALERVG 775
H. species NRC-1      ECRDCG--AVATPDESGRVECPNCGRDLDNVTTQVIDINDEYHGALRAVG 748
A. fulgidus           YCPSCR--MKNTSS-----VCESCGRECEGYMKRKVDLRELYEEAIANLG 717
T. acidophilum        ------------------------HCSPLGEKTGSIDLESILNRAESILG 659

T. species 9°N        VYGI--DRLKGVKGMTSGYKMAEPLEKGLLRVKNDVYVFKDGTIRFDATD 835
T. kodakaraensis      VYGI--DRLKGVKGMTSGYKMAEPLEKGLLRVKNDVYVFKDGTIRFDATD 836
P. furiosus           VYGV--DKLKGVMGMTSGWKIAEPLEKGLLRAKNEVYVFKDGTIRFDATD 825
M. jannaschii         INKP--GDVKCIKGMTSKQKIVEPLEKAILRAINEVYVFKDGTTRFDCTD 760
M. maripaludis        INKP--GDVKCIKGMTSKDKIIEPLEKAILRAKNNIYVFKDGTTRFDCTD 754
H. volcanii           ERESSFQILKGVKGLTSANKTPEPIEKGVLRAKHDVSSFKDGTVRYDMTD 825
H. species NRC-1      ERENAFDQLKGVKGLLSAEKTPEPMAKGVLRAKHDVTAFKDGTVRYDMTD 798
A. fulgidus           EYDS-FDTIKGVKGMTSKTKIPERLEKGILRVKHGVFVFKDGTARFDATD 766
T. acidophilum        ISLDSLKEFKGVKKLMSKEKVAEPIEKGILRAVHDISVNKDGTCRFDMSD 709

T. species 9°N        APITHFKPKEIGVSVEKLRELGYTHDFEGKPLERDDQIVELKVQDIILSY 885
T. kodakaraensis      APITHFKPKEIGTSVEKLRELGYTHDFEGKPLERDDQILELKVQDVILPY 886
P. furiosus           APITHFRPREIGVSVEKLRELGYTHDFEGKPLVSEDQIVELKPQDVILSK 875
M. jannaschii         VPVTHFKPNEINVTVEKLRELGYDKDIYGNELVDGEQVVELKPQDVIIPE 810
M. maripaludis        VPVTHFKPVEIHVPIEKLKSLGYLKDIHGNPLENEDQVLELKVQDVIVPE 804
H. volcanii           LPVTAVRPEELDVTADHFRELGYETDIDGEPLRFDDQLVELKVQDIVLSN 875
H. species NRC-1      LPVTAVTPAELDVTAGQFRELGYNQDIHGDPLEHDDQLVELRVQDVVLSD 848
A. fulgidus           LPITHFKPAEIGVSVEKLRELGYERDYKGAELKNENQIVELKPQDVILPK 816
T. acidophilum        IPITHFRYREIGIDERTLADLGYEVR----------DVNELFPQDVIIPR 749
```

```
T. species 9°N        EAGKYLLKVARFVDDLLEKFYGLPRFYNAEKMEDLIGHLVIGLAPHTSAG 935
T. kodakaraensis      EAGRYLLKVARFIDDLLEKFYGLPRFYNAEKMEDLVGHLVIGLAPHTSAG 936
P. furiosus           EAGKYLLRVARFVDDLLEKFYGLPRFYNAEKMEDLIGHLVIGLAPHTSAG 925
M. jannaschii         SCAEYFVKVANFIDDLLEKFYKVERFYNVKKKEDLIGHLVIGMAPHTSAG 860
M. maripaludis        SCMDYFLNVSGFIDDLLEKYYKKDRFYNVNTREDLVGHLIIGMAPHTSAG 854
H. volcanii           GAAQHMMQTADFVDDLLDQFYGLDRFYEIEERDDLIGELVFGMAPHTSAA 925
H. species NRC-1      GAADHMLKTADFVDDLLTQYYGLDAFYDLDDRDDLVGELVFGMAPHTSAA 898
A. fulgidus           SGAEYLLRVANFIDDLLVKFYKMEPFYNAKSVEDLIGHLVIGLAPHTSAG 866
T. acidophilum        KAAKYLFNVSRFIDDLLVKYYNMPPFYSLESEEDLIGHLIIGLAPHTSGG 799

T. species 9°N        IIGRIIGFSDVLVGYAHPYYHAAKRRNCDGDEDAVMLLLDALLNFSKYYL 985
T. kodakaraensis      IIGRIIGFSDVLVGYAHPYYHAAKRRNCDGDEDAVMLLLDALLNFSKYYL 986
P. furiosus           IVGRIIGFVDALVGYAHPYFHAAKRRNCDGDEDSVMLLLDALLNFSRYYL 975
M. jannaschii         MVGRIIGYTKANVGYAHPYFHAAKRRNCDGDEDSFFLLLDAFLNFSKKFL 910
M. maripaludis        MVGRIIGYSNANVGYAHPYFHASKRRNCDGDEDAFFLLLDAFMNFSKRFL 904
H. volcanii           VVGRVVGFTTAAVGYAHPYFHAAKRRNCDGDEDCVMLLMDGLLNFSKKYL 975
H. species NRC-1      VVGRVAGFTSASVGYAHPYFHAAKRRNCDGDEDCVMLLMDGLINFSKSYL 948
A. fulgidus           VLGRIIGFSDVLAGYAHPYFHAAKRRNCDGDEDCFMLLLDGLLNFSRKFL 916
T. acidophilum        VVGRIIGFSDVNAFYAHPFFHAAKRRNCDGDEDSVMLLMDGFLNFSARYL 849

T. species 9°N        PEKRGGKMDAPLVVTTRLDPREVDSEVHNMDVVRYYPLEFYEATYEMKSP 1035
T. kodakaraensis      PEKRGGKMDAPLVVTTRLDPREVDSEVHNMDVVRYYPLEFYKATYELKSP 1036
P. furiosus           PEKRGGKMDAPLVITTRLDPREVDSEVHNMDVVRYYPLEFYEATYELKSP 1025
M. jannaschii         PDKRGGQMDAPLVLTTILDPKEVDGEVHNMDTMWSYPLEFYEKTLEMPSP 960
M. maripaludis        PDKRGGQMDAPLVLTTILDPKEVDGEVHNMDSMWEYPLEFYEKSLEGIAP 954
H. volcanii           PDKRGGQMDAPLVMSSRIDPSEIDDEAHNMDIVRQYPREFYEATLRMEDP 1025
H. species NRC-1      PDKRGGRMDAPLVMSSRIDPAEIDDEAHNIDIDREYPREFYEATRELADP 998
A. fulgidus           PDKRGGQMDAPLVLTAIVDPREVDKEVHNMDIVERYPLEFYEATMRFASP 966
T. acidophilum        PSTRGGLMDAPLVLSVLINPDEIDKEALNVDTLSRYPVLFYEAAERHASP 899

T. species 9°N        KE-IKFIERVEDRLGKPEMYEGIKFTHDTDDIGLGPKMSLYKQLGDMVEK 1084
T. kodakaraensis      KE-VKVIERVEDRLGKPEMYEGIKFTHDTDDTGLGPKMSLYKQLGDMEEK 1085
P. furiosus           KELVRVIEGVEDRLGKPEMYYGIKFTHDTDDIALGPKMSLYKQLGDMEEK 1075
M. jannaschii         KEVKEFMETVEDRLGKPEQYEGIGYTHETSRIDLGPKVCAYKTLGSMLEK 1010
M. maripaludis        KEIKKMMETIEDRLDKDSQYEGIGYTHETSKIDEGPPICAYKTLGSMMEK 1004
H. volcanii           DDWEDEVTIAEEYLGTDREYTGFDHTHDTTDIAAGPDLSAYKTLGSMMDK 1075
H. species NRC-1      EDVADLITLAESTVGTDEEYTGFGHTHATSNIHLGPSLSAYKTLGSMMDK 1048
A. fulgidus           KEMEDYVEKVKDRLKDESRFCGLFFTHDTENIAAGVKESAYKSLKTMQDK 1016
T. acidophilum        AEIEDTMMTMKVRIKKTGTYMGSSYTMDTSDINSGVLVSSYKTLGTMDEK 949

T. species 9°N        VERQLALAERIRAVDEHHVAETILNSHLIPDLRGNLRSFTRQEFRCVKCN 1134
T. kodakaraensis      VARQLALAERIRAVDEHHVAETIINSHLVPDLRGNLRSFTRQEFRCVKCN 1135
P. furiosus           VKRQLTLAERIRAVDQHYVAETILNSHLIPDLRGNLRSFTRQEFRCVKCN 1125
M. jannaschii         TTSQLSVAKKIRATDERDVAEKVIQSHFIPDLIGNLRAFSRQAVRC-KCG 1059
M. maripaludis        TSAQLAVAKKIRATDERDVAEKVIQSHFVPDLIGNLRAFSRQGVRC-KCG 1053
H. volcanii           MDAQLFLARKLRAVDETDVAERVIEYHFLPDLIGNLRAFSRQETRCLDCG 1125
H. species NRC-1      MDAQLELARKLRSVAETDVAERVIEYHFLPDLIGNLRAFSRQETRCLDCG 1098
A. fulgidus           VYRQMELARMIVAVDEHDVAERVINVHFLPDIIGNLRAFSRQEFRCTRCN 1066
T. acidophilum        INEQLGLAKKLRAVDADDVAARVISTHFLPDMYGNFRKFFSQEFRCTKCN 999

T. species 9°N        TKYRRPPLIGRCPKCGG-KIVLTVSKGAVEKYLPTAKMLVTRYKVKDYTR 1183
T. kodakaraensis      TKYRRPPLTGKCPKCGG-KIVLTVSKGAIEKYLPTAKMLVTKYRVKDYTR 1184
P. furiosus           TKYRRPPLDGKCPVCGG-KIVLTVSKGAIEKYLGTAKMLVANYNVKPYTR 1174
M. jannaschii         AKYRRIPLKGKCPKCGS-NLILTVSKGAVEKYMDVAEKMAEEYNVNDYIK 1108
M. maripaludis        AKYRRMPLKGVCRKCGS-RLILTVSKGAVEKYMNVSQTMAEKYDASDYIK 1102
H. volcanii           EKYRRMPLSGDCRECGG-RVNLTVHQGSVNKYMDTAIQVAEEFDCRDYTK 1174
H. species NRC-1      EKYRRMPLSGDCRECGG-RVNLTVHEGSVNKYMDTAMRVATEYDCREYTK 1147
A. fulgidus           TKYRRIPLVGKCLKCGN-KLTLTVHSSSIMKYLELSKFLCENFNVSSYTK 1115
T. acidophilum        AKYRRIPLSGRCQKCGSTSLTLTIHKGSVVKYLNETLKIAENYRLPDYLK 1049

T. species 9°N        QRICLTEKDIKTLFETVLPEKQRTLLGFSADVCEKMIKERTGSSNGKNGY 1233
T. kodakaraensis      QRICITEKDIKTLFENVFPEKQRTLMGFSADICEKMVKERTGHSNGKNGY 1234
P. furiosus           QRICLTEKDIDSLFEYLFPEAQLTLIVDPNDICMKMIKERTG-ETVQGGL 1223
M. jannaschii         QRLKIIKEGINSIFE----------------------------------- 1123
M. maripaludis        QRLEIIKSGIDSLFV----------------------------------- 1117
H. volcanii           QRLEVLEKSLESVFE----------------------------------- 1189
H. species NRC-1      QRLEIMDRRLESVFE----------------------------------- 1162
A. fulgidus           QRLMLLEQEIKSMFE----------------------------------- 1130
T. acidophilum        ARIDNLARTIKETFP----------------------------------- 1064

T. species 9°N        LDGFNGKNGKAKK----------AKAENEAKREKMPKAEKSKDLK----- 1268
T. kodakaraensis      LDEFNGKNGKASKKSGSLASKLSGKGKEPSKKKESAKPKRSEKVKNLTSF 1284
P. furiosus           LENFNSSGNNGKK---------------IEKKEKKAK------------- 1245
```

```
M. jannaschii         -------NEKSRQ------------------------------------ 1129
M. maripaludis        -------NDKRKQ------------------------------------ 1123
H. volcanii           -------NDKNKQ------------------------------------ 1195
H. species NRC-1      -------DDTNKQ------------------------------------ 1168
A. fulgidus           -------NGTEKQ------------------------------------ 1136
T. acidophilum        -----DTEEEEKPE----------------------------------- 1073

T. species 9°N        ----------------KEVKKEKAKTR-RKRISLDEFFGS- 1291
T. kodakaraensis      EAAAKNEQARGTAGNAKKAESEKPKRKKRKGISLDEFFGS- 1324
P. furiosus           ---------------------EKPKKK--KVISLDDFFSKR 1263
M. jannaschii         -------------------------------VKLSDFFKIG 1139
M. maripaludis        -------------------------------VKIEDFFK-- 1131
H. volcanii           -------------------------------SGIADFM--- 1202
H. species NRC-1      -------------------------------SGISDFM--- 1175
A. fulgidus           -------------------------------VSISDFV--- 1143
T. acidophilum        ----------------------------PREVKITGLDMY- 1085
```

Supplementary Figure 1. Multiple sequence alignment of polD amino acid sequences. (A) The amino acid sequences of polD-S from 9°N, *Thermococcus kodakarensis* (GI:57641837), *Pyrococcus furiosus* (GI:2967437)*, Methanocaldococcus jannaschii* (GI: 499172620), *Methanococcus maripaludis* (GI:45357571)*, Haloferax volcanii* (GI:490144623)*, Halobacterium* sp. NRC-1 (GI:15791200), *Archaeoglobus fulgidus* (GI: 499181746) and *Thermoplasma acidophilum* (GI:499203109) were aligned using ClustalW. Conserved amino acids D507 and H554 in the 3'-5' exonuclease active site that were mutated to alanine are highlighted in black. (B) PolD-L (lacking inteins) amino acid sequences from 9°N, *Thermococcus kodakarensis* (GI:57641838), *Pyrococcus furiosus* (GI:2967437)*, Methanocaldococcus jannaschii* (GI:499173567*), Methanococcus maripaludis* (GI:45047149)*, Haloferax volcanii* (GI:48927395)*, Halobacterium* sp. NRC-1 (GI:15791139), *Archaeoglobus fulgidus* (GI:11499311)*,* and *Thermoplasma acidophilum* (GI:16081956) were aligned using ClustalW. Catalytic aspartate residues (D964 and D966) essential for DNA polymerization (Shen et al. 2001) are highlighted in black. Conserved amino acids with 100% identity (red), 90% identity (green) or 74% identity (yellow) are highlighted.
